# The potential for citizen science to produce reliable and useful information in ecology

**DOI:** 10.1111/cobi.13223

**Published:** 2018-11-27

**Authors:** Eleanor D. Brown, Byron K. Williams

**Affiliations:** ^1^ Science and Decisions Center U.S. Geological Survey 12201 Sunrise Valley Drive Reston VA 20192 U.S.A.; ^2^ The Wildlife Society 5410 Grosvenor Lane, Suite 200 Bethesda MD 20814 U.S.A.

**Keywords:** data quality, ecological science, project design, volunteers, calidad de datos, ciencia ecológica, diseño de proyectos, voluntarios, 数据质量, 生态科学, 项目设计, 志愿者

## Abstract

We examined features of citizen science that influence data quality, inferential power, and usefulness in ecology. As background context for our examination, we considered topics such as ecological sampling (probability based, purposive, opportunistic), linkage between sampling technique and statistical inference (design based, model based), and scientific paradigms (confirmatory, exploratory). We distinguished several types of citizen science investigations, from intensive research with rigorous protocols targeting clearly articulated questions to mass‐participation internet‐based projects with opportunistic data collection lacking sampling design, and examined overarching objectives, design, analysis, volunteer training, and performance. We identified key features that influence data quality: project objectives, design and analysis, and volunteer training and performance. Projects with good designs, trained volunteers, and professional oversight can meet statistical criteria to produce high‐quality data with strong inferential power and therefore are well suited for ecological research objectives. Projects with opportunistic data collection, little or no sampling design, and minimal volunteer training are better suited for general objectives related to public education or data exploration because reliable statistical estimation can be difficult or impossible. In some cases, statistically robust analytical methods, external data, or both may increase the inferential power of certain opportunistically collected data. Ecological management, especially by government agencies, frequently requires data suitable for reliable inference. With standardized protocols, state‐of‐the‐art analytical methods, and well‐supervised programs, citizen science can make valuable contributions to conservation by increasing the scope of species monitoring efforts. Data quality can be improved by adhering to basic principles of data collection and analysis, designing studies to provide the data quality required, and including suitable statistical expertise, thereby strengthening the science aspect of citizen science and enhancing acceptance by the scientific community and decision makers.

## Abstract 2

*Article impact statement*: Well‐designed sampling enhances quality and inferential power of citizen science data and its usefulness for a range of ecological questions.

## Abstract (es)

Potencial de la Ciencia Ciudadana para Producir Información Útil y Confiable en la Ecología

**Resumen**

Examinamos las características de la ciencia ciudadana que influyen sobre la calidad de datos, el poder inferencial, y la utilidad en la ecología. Consideramos temas como el muestreo ecológico (basado en probabilidad, deliberado, oportunista), la conexión entre la técnica de muestreo y la inferencia estadística (basada en diseño, basada en modelo) y los paradigmas científicos (confirmatorio, exploratorio) como trasfondo contextual para nuestra evaluación. Distinguimos varios tipos de investigación de ciencia ciudadana, desde investigación intensiva con protocolos rigurosos enfocados en preguntas claramente articuladas hasta proyectos de participación masiva en plataformas de internet con recolección de datos oportunistas carentes de un diseño de muestreo, y examinamos los objetivos generales, el diseño, el análisis, y la preparación de los voluntarios y el desempeño. Identificamos características clave que influyen sobre la calidad de los datos: los objetivos del proyecto, el diseño y el análisis, y la preparación y el desempeño de los voluntarios. Los proyectos con buenos diseños, voluntarios preparados, y supervisión profesional pueden cumplir con criterios estadísticos para producir datos de alta calidad con un fuerte poder inferencial, y por lo tanto son muy adecuados para los objetivos de investigación ecológica. Los proyectos con una recolección oportunista de datos, un diseño de muestreo ínfimo o nulo, y una preparación mínima de los voluntarios son más adecuados para los objetivos generales relacionados con la educación pública o la exploración de datos ya que la estimación estadística confiable puede ser complicada o imposible. En algunos casos los métodos analíticos estadísticamente sólidos, los datos externos, o ambos, pueden incrementar el poder inferencial de ciertos datos recolectados de manera oportunista. El manejo ecológico, en especial el que realizan las agencias gubernamentales, requiere frecuentemente de datos apropiados para una inferencia confiable. Con protocolos estandarizados, métodos analíticos modernos, y programas supervisados correctamente, la ciencia ciudadana puede contribuir de forma valiosa a la conservación al incrementar el alcance de los esfuerzos de monitoreo para una especie. La calidad de datos puede mejorarse si se adhiere a los principios básicos de la recolección y análisis de datos, se diseñan los estudios para que proporcionen la calidad requerida de datos, y si se incluye una pericia estadística adecuada, fortaleciendo así el aspecto científico de la ciencia ciudadana y aumentando su aceptación dentro de la comunidad científica y con quienes toman las decisiones.

## 摘要

本研究分析了生态学中影响数据质量、推论统计效力和有用性的公民科学的特征。检验的背景包括如生态学抽样 (基于概率的抽样、目的抽样、机会抽样), 抽样技术与统计推断 (基于设计或基于模型) 的联系, 以及科学范式 (验证性或探索性) 等话题。我们区分出不同类型的公民科学调查, 从有清晰明确的问题及严格实验规范的深入研究, 到缺少抽样设计、投机型数据收集的基于互联网的大规模参与项目; 并研究了项目的总体目标、设计、分析、志愿者培训和实现情况。本研究确定了影响数据质量的关键特征, 包括项目目标、设计和分析、志愿者培训和实现情况。拥有良好的设计、训练有素的志愿者和专业监督的项目通常符合统计学标准, 能获得推论统计效力强的高质量数据, 因此可以达到生态学研究目标。而投机型数据收集、很少或没有进行抽样设计且志愿者培训非常有限的项目, 更适合与公共教育或数据探索相关的一般性目标, 因为它们很难或不可能进行可靠的统计估计。在一些情况下, 统计上强健的分析方法和 (或) 外部数据也会增加某些投机型数据的推论效力。生态管理, 特别是来自政府机构的管理, 常常需要那些适合进行可靠统计推论的数据。当采用标准化的实验规范、最先进的分析方法和良好监督的程序时, 公民科学可以扩大物种监测范围, 为保护做出重要贡献。坚持数据收集和分析的基本原则、设计研究方案来提供所需的高质量数据, 并恰当运用统计学知识, 能够增强数据质量, 从而加强公民科学的科学性, 提高科学界和决策者对公民科学的接受度。 **【翻译: 胡怡思; 审校: 聂永刚】**

## Introduction

Citizen science entails the participation of nonprofessional volunteers in scientific investigation. Disciplines including archaeology, astronomy, and biology have long involved amateurs and volunteers, which may be considered citizen science in the broadest sense (Silvertown 2009; Follett & Strezov 2015). Biological projects range from national‐scale projects, such as the U.S. Audubon Society's century‐old Christmas Bird Count (Dunn et al. 2005) and the U.K. Open Air Laboratory (OPAL) surveys of trees, insects, or biodiversity (OPAL 2018), to in‐depth research by small teams, such as those sponsored by the Earthwatch Institute (Brightsmith et al. 2008; Earthwatch 2018). Many other ecological projects use data collected by volunteers (e.g., Devictor et al. 2010; Tulloch et al. 2013; Pocock et al. 2017).

From the name itself, citizen science concerns scientific investigation and citizen engagement. In ecological science, it usually involves the collection of data for comparison with predictions, generation of new hypotheses, or estimation of ecological attributes. Often, projects are specifically designed to give amateurs a role in working with professionals (Silvertown 2009). Citizen science is enthusiastically promoted as increasing public participation in science, while satisfying scientific objectives (Silvertown 2009; Dickinson et al. 2012) and “pushing the envelope of what ecologists can achieve” (Dickinson et al. 2012). Bonney et al. (2009) argue that such projects can “simultaneously fulfill their goals of recruitment, research, conservation, and education.” Some authors (Bonney et al. 2009; Tulloch et al. 2013) hold that citizen science enhances the geographical scope of data collection and provides a source of skills and free labor (Silvertown 2009) that also benefits the public through increased scientific literacy (Bonney et al. 2009).

Nevertheless, the full scientific potential of citizen science remains unrealized (Tulloch et al. 2013; Theobald et al. 2015). It is often viewed skeptically by scientists (Bonney et al. 2014) and has not been used extensively in mainstream ecological research (Theobald et al. 2015) or in management decision making (Conrad & Hilchey 2011). Skepticism by scientists and decision makers is due partly to technical problems such as inadequate consideration of sample size or experimental design (Conrad & Hilchey 2011). A key factor in increasing scientific credibility, as well as the influence and applicability of citizen science data sets, is the enhancement of data quality (Ruiz‐Gutierrez et al. 2016).

We sought to highlight features of citizen science that influence its reliability, inferential power, and usefulness in management and conservation of biological diversity. We view citizen science from the perspective of scientific information, and focus on biological populations, monitoring, and estimation of ecological attributes, through illustrative examples rather than comprehensive review. The scientific aspects of public involvement in ecological research are inadequately treated in the literature, but they are key to scientific practice and deserve a richer discussion.

Our emphasis is on participation of a group (i.e., more than 1) of nonprofessional volunteers in a scientist‐directed, scientific investigation that involves data collection. Framing citizen science in this way is not comprehensive, and many other variants of information collection and analysis are described as citizen science. That said, we focused on projects with a linkage to both scientific process and public engagement.

## Contextual Background for Citizen Science in Ecology

An inferential context for considering public participation in ecological research includes sampling designs and protocols, the strength of inferences that can be obtained from analytic methods, and research paradigms.

### Ecological Sampling

Investigating the distribution and abundance of organisms can require specialized sampling techniques to distinguish real change from natural variation or sampling variability. Sampling can be probabilistic (i.e., random) or nonprobabilistic. In probability‐based sampling, every unit in the population of interest has a known, nonzero probability of being selected, and observations inherit their randomness through the random survey design. In nonprobabilistic sampling, population units are selected without a probability‐based sampling framework. One type of nonprobabilistic sampling is purposive (deliberate) sampling, in which a sampling design targets explanatory factors in a statistical model. Another type is opportunistic sampling, in which chance observations are recorded without a specific sampling design.

Two common problematic issues are nonrepresentative sampling and imperfect detectability (Yoccoz et al. 2001). Nonrepresentative sampling occurs when sampled units are not representative of the population about which inference is to be made (Cochran 1977; Thompson 2012), for example, organisms sampled only along roadsides to estimate density over an area larger than roadsides. Imperfect detectability concerns the lack of detection of organisms when collecting field data. Failing to account for organisms actually present but not detected has obvious consequences for estimating population size, density, and distribution. Nonrepresentative sampling and imperfect detectability can combine with other sampling and observer variability to severely weaken the inferential linkage between ecological attributes and the data collected.

### Statistical Inference

The classical framework for statistical inference in survey sampling is design‐based inference involving probability‐based survey designs (Cochran 1977; Thompson 2012). The possibility that any unit in the population may be sampled establishes the inferential linkage between units that are selected and those that are not. The design allows statistical inferences to be made about an entire population, including the unselected units. Targets of inference with probability‐based sampling include population parameters such as population totals, means, and ratios, which are estimated with the observations (Maas‐Hebner et al. 2015). A strength of design‐based inference is that statistical assessment does not rely on assumed models or data structures. Its main limitations are the restriction of inference to a population of sampling units with nonzero sampling probabilities and a limited ability to address analytical or causal hypotheses (Sterba 2009).

An alternative framework is model‐based inference, which uses statistical models to mimic randomness in the absence of probability‐based sampling (Gregoire 1998; Lenhard 2006). Models typically include parameters for hypothesized factors (e.g., variable observer effort, nondetectability, habitat variation) that are thought to influence observations. The assumed applicability of models to all potential observations allows inference to be extended beyond the sample to the population. An important limitation is that the data collected may not fully represent the population of interest because the survey design is not probability based (Maas‐Hebner et al. 2015).

Model‐based inference can potentially be applied to data collected through purposive or opportunistic sampling. Purposive sampling is linked directly to a model, and data are collected deliberately to strengthen inferences about population parameters. With opportunistic sampling the linkage is missing, and the data may or may not prove useful for reliably estimating parameters. In either case the models include covariates that are hypothesized to influence observations, and analysis involves estimating covariate parameters with the sample data. This approach may produce reliable inferences if the relevant environmental and observer factors are accounted for in the model, and the necessary covariate information about them is collected (Sterba 2009). However, with opportunistically collected data the joint requirements of model specification and data collection are rarely met, and other information (from metadata, external data sources, follow‐up sampling) is needed to improve statistical reliability.

Much knowledge that is critical for conservation, such as absolute abundance or density, can be obtained with a probability‐based sampling design and standardized protocols for inference about a population of interest. In addition, important information about rate parameters such as reproduction and mortality rates can be obtained through model‐based inference from data collected purposively. These approaches contrast with opportunistic data collection without any sampling design, which is unlikely to result in reliable inferences about a target population without information beyond the survey.

### Confirmatory Versus Exploratory Science

John Tukey (1977, 1980), a pioneer in statistics, distinguished between exploratory and confirmatory research paradigms and how each affects hypothesis formation, study design, data collection, and analytical methods. In the confirmatory paradigm, hypotheses are generated a priori, before data collection, whereas in the exploratory paradigm hypotheses are generated a posteriori, after collection. Confirmatory analysis can provide reliable scientific information, through the investigation of well‐defined a priori hypotheses with statistical analysis of data collected according to designs that target the hypotheses. Alternatively, exploratory analysis of data can be useful in formulating hypotheses a posteriori in a 2‐step process: exploration of data (often collected opportunistically) for patterns that lead to hypotheses, and then design of new investigations that can test whether the patterns genuinely express underlying ecological structure. The statistical reliability of results cannot stand on the exploration alone without some form of follow‐up study.

Ecology involves both exploratory and confirmatory analysis, depending on the circumstances. Nonetheless, it is important not to confuse one with the other. The distinction between them is particularly relevant to citizen science, which often involves opportunistic collection of large amounts of data and exploratory analysis with new Web 2.0 technologies (Goodchild 2007).

## Project Features Influencing Data Quality and Usefulness

Several factors influence the quality and usefulness of data for estimation and inference, particularly in population research and monitoring.

### Typology of Citizen Science Projects

In our typology, overall design of projects ranges from rigorous protocols designed for clearly articulated questions to opportunistic data collection without protocols. Our grouping builds on the pyramid framework of the U.S. Geological Survey's Amphibian Research and Monitoring Initiative (Corn et al. 2005): intensive research at handpicked sites at the top, standardized monitoring with rigorous sampling design over a broad area in the middle, and coarse measurements in a checklist approach at a national scale at the bottom. We used a similar typology, recognizing considerable variation within and among groups (Pocock et al. 2017).

In intensive scientific research projects, volunteers work in small teams under close supervision by lead scientists. Volunteers may perform complex tasks and collect data according to explicit protocols that often allow confirmatory hypothesis testing and statistical inference. Examples include projects sponsored by the Earthwatch Institute (Earthwatch 2018), a nongovernmental organization that connects ecotourists with conservation‐oriented research (e.g., Peruvian rainforest birds [Brightsmith et al. 2008]).

Longitudinal monitoring studies consist of standardized repeat monitoring, often with many participants and a high degree of institutional coordination. Data on species occurrence and relative abundance may be collected by volunteers; sampling protocols involve specified time intervals and locations (Tulloch et al. 2013), allowing confirmatory hypothesis testing and statistical inference. Some level of biological expertise is frequently a prerequisite. This category includes surveys such as the British (Newson et al. 2005), Swiss (Kéry & Schmidt 2004), and North American (Sauer et al. 2013) Breeding Bird Surveys.

Atlas‐type monitoring studies involve a substantial number of participants and a rudimentary sampling scheme, entailing data collection over a defined time period, in a grid of broadly defined spatial cells within which volunteers can choose locations (Tulloch et al. 2013). Data may consist of lists of species occurrence or checklists, sometimes designed to yield relative abundance within grid cells. An atlas can be a single survey of species spatial distribution, or can include repeat sampling. Repeat‐visit data and statistical adjustments may allow model‐based inferences and unbiased estimates of quantitative population trends (e.g., Link et al. 2006). One example of a repeat‐visit atlas is the U.S. Audubon Society's annual Christmas Bird Count (Dunn et al. 2005), among many other atlases (Tulloch et al. 2013).

Finally, internet‐based projects that accept opportunistic data on open‐access web portals may involve no sampling scheme, few or no data‐collection protocols, little or no training, and mass participation. Typically, presence‐only observational data (location, occurrence of species) collected opportunistically are recorded via web portals, often covering broad areas. Potential bias is frequently induced by volunteer skill differences, spatial variation, and other factors (Isaac et al. 2014). Analysis often requires additional statistical modeling with assumptions that can constrain use of the data, and it rarely produces reliable estimates without follow‐up investigation. This category includes the Swedish Species Gateway bird‐monitoring project (Snäll et al. 2011), a country branch of Worldbirds (Roberts et al. 2005); projects led by the Cornell Lab of Ornithology (Bonney et al. 2009; Dickinson et al. 2010; Hochachka et al. 2012); and the Australia‐based QuestaGame project (QuestaGame 2018).

## Project Objectives

Objectives that guide project design and data collection influence data quality. Objectives of applied ecological research can be pursued in projects with strong designs, expertise and training of volunteers, and professional oversight. Such projects may produce long‐term data usable for reliable estimation involving multiple research questions. An example is the North American Breeding Bird Survey, a longitudinal survey that targets various information needs for species of conservation concern such as Neotropical migrants and grassland birds (Sauer et al. 2013).

However, many large‐scale internet‐based projects with opportunistic data collection are better suited for general objectives related to public education, recreation, or data exploration (e.g., Bonney et al. 2009; Tulloch et al. 2013). For these purposes it is unnecessary to meet the temporal replication and other design requirements needed to distinguish real ecological patterns from error or natural variation. Large‐scale projects with opportunistic data collection often involve surveillance monitoring of numerous species over a broad area with no specific a priori research questions (Dickinson et al. 2010), unlike targeted monitoring that is designed to investigate particular questions or models (Nichols & Williams 2006). Rather than hypothesis testing and statistical estimation, data exploration is often used to search for potential patterns, such as species occurrence and habitat associations (Hochachka et al. 2012).

## Project Design and Analysis

As with all investigations, design can govern the questions addressed, data collected, and analytical methods (Williams et al. 2002; Bird et al. 2014). For survey and monitoring design in general, factors including the number of taxa, number of sites, sampling variability, sampling frequency, and duration of monitoring are major determinants of the analysis techniques that can be used for reliable inference. In many respects a gold standard of survey design entails data for presence and absence or abundance; stratified random sampling across a geographic area; standardized protocols that control for sources of bias; and metadata that allow quantification of error (Yoccoz et al. 2001; Magurran et al. 2010; Isaac et al. 2014).

Designed approaches to inference can be used with hypothesis‐driven projects and longitudinal surveys to accomodate some or all of the foregoing standards, particularly in basic and applied research requiring high‐quality data (e.g., Williams et al. 2002). Good design can account for the 2 main sources of variation in monitoring data (Yoccoz et al. 2001) through spatial sampling that allows for reliable representation over an area to which inference is made, and temporal sampling that allows quantitative estimation of detection probability (Yoccoz et al. 2001; Buckland et al. 2005; Magurran et al. 2010). Databases from such projects are often usable to address a wide range of questions.

For many atlas‐type projects and projects with open‐access internet contribution of opportunistically collected data, reliable estimation and confirmatory testing of a priori hypotheses may be difficult or impossible. Such projects may not produce the sample data needed to address high rates of variation in visit frequency, observer effort, or species misidentification (Isaac et al. 2014). Other common sources of variation result in nonrepresentative sampling of geographic areas. Additional statistical assumptions and data constraints can limit the range of research questions that can be addressed with such data sets (Nichols et al. 2012).

With some opportunistically collected data, statistically robust analytical methods or external data may increase the inferential power of a model‐based approach. Statistical adjustments can sometimes reduce variability and bias and compensate for data features that violate statistical assumptions. For example, occupancy modeling (MacKenzie et al. 2017) can provide unbiased information on temporal trends in opportunistic data sets that meet certain conditions for temporal replication, consistent data‐collection methods, and covariate information (Kéry et al. 2010; van Strien et al. 2013; Isaac et al. 2014). In other examples, Kindberg et al. (2009) and Szabo et al. (2010) used visit duration and list length, respectively, as proxies for survey effort in monitoring large mammals and birds. Maes et al. (2015) discussed methods for estimating species distribution and geographic range from opportunistic data sets for International Union for Conservation of Nature (IUCN) Red List purposes. Bird et al. (2014) reviewed modeling techniques.

Although exploratory analysis of patterns in data collected opportunistically is always possible, it is still necessary at a minimum to deal with survey effort and detection issues. Otherwise the patterns that are discovered may confound sampling error with ecological processes.

## Volunteer Training and Performance

A further factor influencing data quality is volunteer training and performance tracking. Some volunteers, amateur or otherwise, may already be subject‐matter experts, and with sufficient training many can perform some tasks as well as professionals. However, the more complex the task, the greater the investment needed in training and professional supervision (Foster‐Smith & Evans 2003; Newman et al. 2003). The hands‐on training and oversight offered by intensive research teams is necessary for teaching complicated tasks (e.g., trapping, point counts for woodland mammal monitoring [Newman et al. 2003]; global positioning systems, forest inventory plots for invasive plant monitoring [Crall et al. 2011]; taxonomy of Hymenoptera for pollinator identification [Kremen et al. 2011]). The more limited training needed for broad‐scale internet‐based projects involving simple tasks (identifying a small number of species, counting eggs) may be provided as online instructional materials and quizzes (e.g., Bonney et al. 2009), sometimes given as feedback in conjunction with data entry and screening of data for obvious errors. Some opportunistic monitoring projects may provide no training of any kind (e.g., Roberts et al. 2005).

Observer bias in citizen science projects has been investigated by experimentally comparing data collection by volunteers and professionals. Data reliability varies depending on the volunteer group, the species, the ecosystem, and the task (Steger et al. 2017). In many studies, volunteers were comparable to professionals in straightforward identification of species or higher‐level taxonomic groups (insect pollinators [Kremen et al. 2011], amphibians [Genet & Sargent 2003], woodland mammals [Newman et al. 2003]; but see Roy et al. [2016]); although not in making difficult taxonomic identifications (coccinellid lady beetles [Gardiner et al. 2012], calling amphibians [Genet & Sargent 2003], insect pollinators [Kremen et al. 2011]). In detecting organisms, volunteers were comparable in some cases (mammals on territories [Newman et al. 2003]), but not if expertise comes from lengthy experience (cryptic marine invertebrates [Foster‐Smith & Evans 2003], nocturnal mammals [Sunde & Jessen 2013], African ungulates in herds [Steger et al. 2017]). Of course, professionals frequently fail to detect organisms, too. The solution is survey design allowing quantitative estimates of detection error (Williams et al. 2002). More generally, addressing observer bias requires monitoring performance and accounting for data variability with metadata (Crall et al. 2011; Milner‐Gulland & Shea 2017).

## Discussion

Project design, rather than citizen involvement per se, is a fundamental constraint limiting the use of citizen science data in ecological research. The issue is how data are collected, not by whom (professionals or volunteers). Intensive research or longitudinal monitoring studies, designed around a priori hypotheses or defined questions, often involve more expense and labor, such as investment in training and supervising volunteers, but allow for stronger inference and ensure that data collected are well‐suited for their intended use (Nichols et al. 2012). We emphasize that testing of ecological hypotheses in the confirmatory paradigm is entirely possible with large‐scale, internet‐based citizen science projects if they are specifically designed for that purpose, although such projects are rare. Examples include studies by Silvertown et al. (2011) of evolution of snail color morphs and by Pocock and Evans (2014) of parasitism of an invasive leaf‐miner, both projects involving simple protocols suitable for mass participation.

In contrast, observational approaches lacking the necessary design features for strong inference may be less expensive, but require data adjustments and additional analytical assumptions that can limit inferences (Nichols et al. 2012). Internet‐based projects involving mass contribution of opportunistic data by participants at all skill levels (e.g., Bonney et al. 2009) often produce data that lack strong inferential power and require exploratory searching for patterns in order to generate hypotheses for follow‐up investigation. As mentioned, the combined requirements for model specification and data coverage in a model‐based approach limit the use of opportunistic data sets for reliable inferences, although external or covariate information may compensate (Kéry et al. 2010; van Strien et al. 2013; Isaac et al. 2014). Similarly, these conditions may allow some atlas‐type studies to produce reliable inferences (e.g., Dunn et al. 2005; Link et al. 2006; Tulloch et al. 2013).

Many citizen science participants aspire to actionable science that decision makers can use for management, planning, and stewardship (Conrad & Hilchey 2011; Ganzevoort et al. 2017). Project planners with these goals must take into account the importance of data quality for decision makers, especially in government. Much ecological research carried out by agencies (e.g., U.S. Department of the Interior 2018) is applied research to meet ecological management or policy objectives, including conservation. This frequently requires high‐quality data suitable for reliable estimation and inference, with which management‐related ecological hypotheses can be tested or the effects of management actions can be distinguished from sampling error and natural variation.

Two conservation‐related examples of such a management context are species recovery under the U.S. Endangered Species Act and invasive species control, which can be very costly. Such regulatory responsibilities may have exacting needs for data quality (Johnson et al. 2015), and land managers need assurance of quality before they embark on expensive programs (Newman et al. 2010). To meet such needs, citizen science project methods must be chosen carefully regarding the level of accuracy and access to statistical expertise for specific research questions (Crall et al. 2011). Well‐designed intensive research studies and longitudinal monitoring studies are usually best suited. Volunteer training and regular performance monitoring are essential in establishing a record of reliability (Crall et al. 2011), as is accounting for sources of data variability (Pocock & Evans 2014; Steger et al. 2017).

For biodiversity conservation, 1 way citizen science can potentially make valuable contributions is by increasing the number of observers and geographic scope of species monitoring efforts. With standardized protocols, state‐of‐the‐art analytical methods, and a well‐supervised program, data from nonprofessionals can provide reliable results (Magurran et al. 2010; Schmeller et al. 2009; Tulloch et al. 2013). Citizen‐science investigations could be used in conservation biogeography to examine large‐scale patterns and processes, for example the impacts of climate change on biodiversity (Devictor et al. 2010). Buckland et al. (2005) suggest designing longitudinal monitoring schemes in common across global regions, with surveys entered at various levels (modified by lower sampling rates or simpler methods) so nations with fewer resources could participate. Standardized citizen science monitoring schemes could also be used for population trend estimates necessary for IUCN Red List assessments of rare species (Maes et al. 2015) or to track spatial dynamics of invasive species (Eraud et al. 2007). For volunteers, participation can foster new skills and enthusiasm for science (Devictor et al. 2010). For example, in an Earthwatch project surveying British woodland mammals, 30% of volunteers joined conservation groups, and 5% actually changed careers to biology (Newman et al. 2003).

In some instances, historical opportunistic data sets can yield reliable analysis of temporal trends, as long as robust methods can be used with model‐based approaches to increase the inferential power of opportunistic data (van Strien et al. 2013; Isaac et al. 2014). This can be an important source of information about changes in species relative abundance or distribution. For example, historical opportunistic data sets were used by van Strien et al. (2018) to examine whether woodland fungi benefited from policies that reduced atmospheric nitrogen and by Sparks et al. (2005) to examine the association between warming temperatures and abundance of butterflies migrating to Britain.

In sum, measures for strengthening the science aspect of citizen science in ecology will increase its acceptance by scientists and decision makers. Core issues such as the limits of what we can expect from public contributions should be more widely discussed, along with methodological questions regarding data quality (Bird et al. 2014; Riesch & Potter 2014). Basic principles of data collection and analysis (Theobald et al. 2015) are essential for science quality and management relevance. Study methods need to accord with the purpose of the data (Steger et al. 2017). If the idea is to use citizen science data for enhancing public input into environmental governance (e.g., Conrad & Hilchey 2011), or for the policy relevance desired by Dickinson et al. (2010), the study design should ensure that the defined standards of data quality can be achieved (Haklay 2010) for the particular ecological management context. Statistical expertise, data treatments, and constraints on sampling (Bird et al. 2014; Isaac et al. 2014) should be factored into the study from the beginning. Collecting data to deal with imperfect detection (e.g., by using temporal replication), a practical reality for volunteers and professionals alike, is especially important.

In some cases, project objectives related to social research, public participation, and scientific literacy may be more important than objectives involving hypothesis confirmation in basic or applied research. For example, U.S. National Science Foundation programs that provide funding for projects (e.g., Bonney et al. 2009; Hochachka et al. 2012) in computer science or informal science learning explicitly include goals of public access to science learning opportunities (National Science Foundation 2018*a*) and to “affordable participation in an information‐based society” (National Science Foundation 2018*b*). Objectives such as public engagement often do not require statistical inference, so the limited data requirements can be met with less‐rigorous design or sampling protocols (Tulloch et al. 2013). In ecological investigations, in contrast, improved quality and usefulness of data collected by volunteers will help strengthen inferences and ensure results from citizen science are accepted and used appropriately (Tulloch et al. 2013).
